# Heterogeneity in treatment outcomes and incomplete recovery in first episode psychosis: does one size fit all?

**DOI:** 10.1038/s41398-022-02256-7

**Published:** 2022-11-17

**Authors:** Siân Lowri Griffiths, Paris Alexandros Lalousis, Stephen J. Wood, Rachel Upthegrove

**Affiliations:** 1 Institute for Mental Health, University of Birmingham, Birmingham, UK; 2 Centre for Human Brain Health, University of Birmingham, Birmingham, UK; 3 Orygen, the National Centre of Excellence in Youth Mental Health, Melbourne, VIC Australia; 4 Centre for Youth Mental Health, The University of Melbourne, Parkville, VIC Australia; 5 Birmingham Early Interventions Service, Birmingham Women’s and Children’s NHS Foundation Trust, Birmingham, UK

**Keywords:** Schizophrenia, Scientific community

## Abstract

The heterogeneity in recovery outcomes for individuals with First Episode Psychosis (FEP) calls for a strong evidence base to inform practice at an individual level. Between 19–89% of young people with FEP have an incomplete recovery despite gold-standard evidence-based treatments, suggesting current service models, which adopt a ‘one-size fits all’ approach, may not be addressing the needs of many young people with psychosis. The lack of consistent terminology to define key concepts such as recovery and treatment resistance, the multidimensional nature of these concepts, and common comorbid symptoms are some of the challenges faced by the field in delineating heterogeneity in recovery outcomes. The lack of robust markers for incomplete recovery also results in potential delay in delivering prompt, and effective treatments to individuals at greatest risk. There is a clear need to adopt a stratified approach to care where interventions are targeted at subgroups of patients, and ultimately at the individual level. Novel machine learning, using large, representative data from a range of modalities, may aid in the parsing of heterogeneity, and provide greater precision and sophistication in identifying those on a pathway to incomplete recovery.

## Heterogeneity in illness trajectories and outcomes

### Recovery outcomes in early psychosis following early intervention service: does one size fit all?

The traditional Kraepelinian view posits that schizophrenia is defined by enduring impairment and inevitable decline. We now know that treatment response is positive for many young people with First Episode Psychosis (FEP), particularly since the introduction of Early Intervention Services (EIS) in the late 1990s [1].

EIS offers specialised assertive outreach care during a hypothesised ‘critical period’, which represents the first 3–5 years following illness onset, as well as aiming to reduce the period of untreated psychosis, where illness progression is rapid [2]. Appropriate intervention during this time is shown to have the greatest impact [3]. Indeed, it is established that EIS leads to improved functional and clinical recovery compared to standard community care [1].

Despite ‘gold-standard’ treatment under EIS, systematic reviews provide evidence of substantial heterogeneity in recovery outcomes for young people with FEP. Incomplete symptomatic recovery rates range between 19–89%, but there is also significant heterogeneity of non-recovery for social and vocational functioning, ranging between 46–86% [4, 5]. These findings suggest that current EIS service models may not be addressing the needs of all young people with early psychosis. This calls for better identification of individuals at risk of poor recovery, and appropriate tailoring of interventions.

### Challenges to predicting recovery outcomes in early psychosis

Predicting outcomes in FEP is complex. Firstly, the concept of ‘recovery’ lacks a clear definition and there are no ‘gold-standard’ measures to comprehensively assess recovery; this will in part account for the variability in recovery rates reported across studies [6]. Whilst there is no consensus on what recovery should entail, the concept encompasses broad dimensions, with a distinction being drawn between personal recovery, involving subjective quality of life irrespective of symptoms and functioning, and clinical recovery, which is often based on observer-rated outcomes of symptomatic remission and adequate functioning over a specified timeframe [6, 7]. Recovery is therefore a multidimensional concept that is likely to be multidimensionally determined. This makes predictions at the individual level challenging, particularly in the early stages of psychosis where illness trajectories are forming and the clinical picture is still emerging [8].

There is also considerable variance in defining treatment resistance, which has primarily been associated with unremitted positive symptoms [9, 10]. This fails to capture other important symptoms such as cognitive impairment and negative symptoms, which are largely unresponsive to standard treatments [11–14]. It also does not consider poor social and role functioning, which may persist even when symptomatic improvement occurs [15].

An international consensus group have made recommendations for broadening optimal criteria for treatment resistance in schizophrenia, which includes a) persisting symptoms of at least moderate severity, b) moderate or worse functional impairment, c) a trial of two sequential antipsychotics at a therapeutic dose, d) systematic monitoring of treatment adherence [9]. Presently, there is no international consensus on defining emerging treatment resistance following FEP; this would be pertinent given that diagnoses are more fluid and positive symptoms are often more responsive in this early stage of illness [16, 17]. Incomplete recovery has been suggested as an alternative term, which considers persisting impairment in psychosocial as well as functional domains despite evidence-based treatments; a concept that also signifies the prospect of improved therapeutic outcomes [10, 18].

Despite the shortcomings in defining and assessing these concepts, a number of demographic, illness-related, and treatment-related factors are shown to predict incomplete recovery in early psychosis, which include: long delay in untreated psychosis (DUP), younger age at onset, cognitive impairment, negative symptoms, affective comorbidity, substance use, treatment non-adherence, initial treatment response, and male gender [13, 14, 19–22].

We next discuss how some of these factors pose challenges in the delineation of heterogeneity in early psychosis, and whether current approaches and service models are adequate in addressing these disparities.

### Initial treatment response

In the UK, NICE recommends antipsychotic medication and psychological interventions such as Cognitive Behavioural Therapy (CBT) and Behavioural Family Therapy (BFT) as first line treatments for all individuals with psychosis, but there is considerable heterogeneity in response to these evidence-based treatments [23]. Only 20% of people with psychosis experience full response to pharmacological treatment, with the remainder experiencing partial response [24, 25]. Initial response to treatment is one of the strongest predictors of longer-term outcomes; 70% of those who are deemed treatment resistant are treatment resistant from illness onset [19, 26]. This presents an opportunity to identify those on a pathway to incomplete recovery from illness onset (i.e. those with early treatment resistance), and intervene appropriately to prevent entrenchment of symptoms.

However, it is important to establish if incomplete recovery is owing to a lack of adherence to medication [27]. In relation to symptomatic recovery, sustained use of antipsychotic medication is associated with reduced likelihood of relapse; this is important as further relapses are associated with diminishing treatment response, as well as greater distress and economic cost [28, 29]. In contrary, there is also evidence suggesting prolonged antipsychotic use at a high dose is negatively associated with recovery outcomes, particularly with regards to cognitive function and social and vocational functioning [30–32]. Robust evidence is needed to understand the impact of dose reduction on improving cognitive and functional outcomes in stable patients with FEP [33, 34]. This would provide greater knowledge for the clinician and young person to carefully navigate the associated risks and benefits of dose reduction vs. continuing higher doses of antipsychotic medication [34].

For those without symptomatic remission, clozapine is the only evidence-based psychopharmacological therapy for treatment resistance, and the recommendation is for the commencement of clozapine following a trial of two unsuccessful standard antipsychotics [35]. In a recent study exploring prescribing practices across UK EIS, a clear stasis in treatment was evident for those who are treatment resistant and eligible for clozapine [17]. For example, there was a tendency for polypharmacy, where these individuals continued to be prescribed antipsychotics despite a lack of improvement [17]. This may suggest a missed opportunity to influence recovery during the ‘critical period’ and given the superior efficacy of clozapine in reducing suicide risk, in addition to improving symptomatic as well as functional recovery, this missed opportunity may have significant consequences [17, 36, 37].

There is also considerable heterogeneity in response to psychosocial interventions in psychosis, and standard CBT for psychosis is less likely to be effective for subgroups of individuals with complex illness presentations [38–40]. Baseline factors such as cognitive impairments are shown to have a rate-limiting impact on treatment outcomes [41–43]. Thus, to ensure interventions are being delivered appropriately, greater precision in identifying individuals who are likely to benefit is needed, along with an understanding of the mechanistic markers of change that would enable the process of early treatment stratification [43].

### Diagnostic uncertainty and comorbidity

Diagnostic ambiguity in the early stages of psychosis presents a challenge for establishing the course of illness and appropriate treatment. In a study looking at diagnostic stability in FEP, 54.2% were assigned the same diagnosis from first presentation to first year follow-up, but this increased to 95.7% by the second year, suggesting that symptoms may not fully manifest within the first year of treatment [44]. Despite a lack of stability in the early stages of illness, even within diagnostic categories, no two patients present with the exact same constellation of symptoms, calling into question the validity of a categorical approach for understanding and managing mental illness [45, 46].

Comorbid symptoms have also contributed significant debate regarding heterogeneity in recovery outcomes in FEP, their function as prognostic indicators, and their place within hierarchical diagnostic structures. In particular, affective comorbidities such as depression are highly prevalent in FEP and are associated with poorer recovery [14]. At least a quarter of FEP individuals meet full diagnostic criteria for a depressive disorder, and nearly half of this population will experience significant depressive symptoms warranting intervention [47]. Depression is the most significant risk factor for suicidal behaviour in FEP and has long-term consequences for other poor outcomes, including social and occupational recovery, increased risk of substance misuse, medication non-adherence, and reduced quality of life [27, 48].

Despite the established relationship between psychosis, depression, and poor recovery outcomes, the diagnosis and management of this comorbidity presents a continued problem for clinicians, with a reported rate of misdiagnosis of over 27% [49, 50]. Moreover, there are no large-scale controlled trials investigating the effectiveness of adjunctive antidepressants, or CBT, to specifically target depression within psychosis [14, 51, 52]. Improved recognition and management of depression to improve recovery within EIS is needed [49].

Depressive symptoms are highly interrelated with both positive and negative symptoms of psychosis and might therefore be best conceptualised as intrinsic to FEP [53, 54]. Though the Kraepelinian dichotomy has long been challenged, current nosology and diagnostic frameworks still classify depression and schizophrenia as separate disease entities. More recently, research adopting a novel network perspective has demonstrated a significant interplay between clusters of symptoms over the development of disorder through to onset; these symptoms may interact to maintain psychopathology [54, 55]. If symptoms such as depression are identified correctly, novel symptoms may be new targets for effective treatments [53].

Substance use is also likely to influence recovery outcomes. Persistent cannabis use is associated with higher relapse rates, prolonged hospital admissions, poor functional outcomes, and more severe positive symptoms compared to non-users [22, 56]. In a ten-year longitudinal study, recovery outcomes for those who discontinued cannabis use prior to the follow-up, resembled that of the non-users, suggesting that the impact of psychosis may be reversed upon cessation of consumption [56]. Offering therapeutic intervention for cannabis cessation earlier in the illness course may promote recovery. Furthermore, there is strong evidence for an association between persistent cannabis use and earlier age of onset of psychosis; reducing the use of cannabis might be one modifiable way to delay, or even prevent the onset of the disorder [57]. Further work should seek to explore these temporal relationships.

### Delay of untreated psychosis

DUP is shown to predict a wide range of outcomes after FEP, including persistence of positive and negative symptoms, cognitive function, and poor social outcomes [58]. Investigation of DUP has contributed significantly to understanding the importance of timely interventions for more favourable outcomes. Generally, longer DUP (>6 months) is associated with incomplete recovery, but in a longitudinal modelling study by Drake and colleagues, they showed a curvilinear relationship between DUP, symptom severity, and treatment response in FEP. Refractory symptoms were associated with a longer DUP, however, this response was initially more rapid and then plateaued [20]. This further highlights the importance of prompt access and initiation of effective treatments ideally within the first few weeks of illness onset [20].

Whilst the implementation of EIS focuses efforts on the reduction of DUP, many individuals continue to have a DUP exceeding 6 months [58–60]. Further, a meta-analysis of controlled trials aiming to reduce DUP, which included stand-alone FEP services, high risk services, public health initiatives, and multi-focus interventions, provided no summary evidence of reducing DUP in FEP [58]. Robust, large-scale initiatives are likely needed to test the efficacy of suitable interventions, but this will be difficult to achieve given the sample size and power required to show a significant reduction in incidence rates [61]. Indicated prevention where vulnerable groups are targeted with preventative strategies may be more pragmatic and effective, but this would require greater specification of the markers of psychosis risk.

### Social determinants and the role of the environment in the pathogenesis of psychosis

There are many social and environmental factors which are heavily implicated in the development of psychosis, and these are also likely to operate within the process of recovery [8, 18]. Urbanicity, pervasive disadvantage, minority status, marginalisation, and childhood adversity are key drivers, which if not addressed, are likely to perpetuate a cycle of disadvantage and hinder the recovery process [62–64]. A better understanding of how these factors interact to predispose these individuals to illness and poor outcomes is needed, and these individuals may also be a candidate group for indicated prevention.

There is now a growing interest in the mechanisms by which environmental factors can have impact on the pathogenesis of psychosis. The immune hypothesis of psychosis proposes that exposure to early adversity or stressful environments may ‘prime’ the brain for the development of psychotic disorders [65]. Cytokines, which are markers of inflammation, are involved in early brain development; perturbations of cytokines (induced by stressful environments) during critical periods of neurodevelopment may increase psychosis liability [66]. Indeed, childhood adversity, which increases risk to psychotic disorder by two-fold, is associated with elevated levels of proinflammatory cytokines [67]. There is also evidence of chronic low-grade inflammation in a subgroup of individuals with early psychosis, which is linked with cognitive impairments and negative symptoms [68, 69]. Integrating neurobiological and psychosocial factors may help to identify more robust and sensitive markers or identify specific disease phenotypes within this heterogenous group, potentially providing new treatment avenues.

### Parsing heterogeneity and moving towards stratified intervention approaches

It seems apparent that there are subgroups within FEP who do not make equal gains in their recovery despite receiving high quality EIS care, adopting NICE approved psychosocial and pharmacological interventions [18]. Earlier, stratified interventions are urgently needed to ensure all individuals can achieve equitable gains to maximise their life chances.

The availability of large data now enables novel mathematical modelling to delineate complexity and heterogeneity within mental illnesses. Machine learning—a branch of artificial intelligence—holds significant potential in overcoming the difficulties faced by traditional statistics in mental health research [70]. For example, classic inferential approaches seek to reject the null hypothesis by considering the entirety of a data sample rather than focusing on individual cases or subgroups [71]. The reproducibility and generalisability of traditional approaches are also increasingly scrutinised [71].

### Machine learning

Machine learning methods rely on few assumptions and allow for the mining of structured knowledge from extensive data that can be applied to individual cases, allowing for personalised approaches in mental health diagnosis and treatment [70].

Data from different modalities (e.g. clinical, genetic, biological and neuroimaging data), can be incorporated into a prediction model to identify different illness phenotypes and mechanistic markers which could potentially inform more objective nosology and improve prognostic certainty [18, 72, 73]. The prognostic potential of machine learning has so far been demonstrated in early psychosis studies predicting symptomatic and functional recovery [4, 73]. More recently, this has extended to transdiagnostic predictors of poor recovery across disorders. For example, our group has demonstrated transdiagnostic features which are likely to be intrinsic to early psychosis [74]. We identified that disease processes in psychosis with comorbid depression align more strongly with a depressive prototype [75] and showcased two neuroanatomically based transdiagnostic clusters which could improve development of stratified treatments and identification of poor outcome patients [76]. This could pave the way to better develop targeted adjunctive treatments and better outcomes for comorbid, and complex presentations.

Finally, the way in which interventions are currently evaluated are based on the average patient, and therefore do not sufficiently capture the heterogeneity in treatment response. The utility of machine learning may also be applied within a clinical trial design to identify subgroups of individuals who do not respond, as well as markers of non-response, allowing for the stratification and development of new treatments for these individuals [18].

### Model translation and challenges

The evidence base for the clinical application of machine learning to everyday clinical practice is growing, with the potential to inform objective diagnoses, illness prognoses, and stratified treatments [72]. For example, by utilising data from medical records, individuals at risk of intensive care use have been identified using machine learning [77]. From a diagnostic perspective, machine learning trained on imaging data has shown to enhance detection of diminutive adenomas and hyperplastic polyps [78]. Finally, with the application of machine learning algorithms, treatment response to fifteen distinct cancer types has been achieved [79]. These findings are promising for application of machine learning into psychosis care and treatment, but as the field moves towards translation, there a number of considerations.

Challenges to integrating machine learning into everyday practice include the lack of availability of technologies to apply data from a range of modalities [72]. Second, machine learning requires specialist knowledge and skills to interpret accurately, which adds to the burden on the busy clinician. Moreover, it is imperative that test data represent real-world sample heterogeneity so prediction models can accurately inform the care of the individual [71]. This would require large and diverse datasets, which are often not available, possibly leading to minority groups being underrepresented in clinical research, which undermines the generalisability of the models [72]. As we’ve previously highlighted, the complexity of recovery extends beyond the clinical picture and may mirror the inequity in our health systems and social structures for disenfranchised groups [18, 80, 81]. A better understanding of how these predisposing factors continue to drive illness course is imperative, but so far, few multimodal approaches include environmental and societal factors into machine learning models in early psychosis [62, 82].

Finally, considerations should be given to the ethical implications associated with the translation of machine learning into routine health care [83]. There may be concerns around data surveillance from the gathering of personal data from devices, which overlooks the autonomy of the individual [84]. Furthermore, given the history of funding cuts to mental health services, there is a risk that certain individuals who are deemed by an algorithm to have better prognoses, may, as a result, receive reduced care [85]. Similarly, there is also a view that machine learning will be used for defensive purposes—for example, suicide risk calculators—rather than being used to best serve the needs of the patient group. Finally, information from prediction models should be used as a guide for clinical decision making, not to replace it, and nor should it replace the individual’s narrative in person centred care [18].

## Conclusions

There exists considerable heterogeneity in outcomes after FEP, and prediction at the individual level is currently very challenging. The application of machine learning may hold promise in parsing these features and delineating the complexity to improve prognostic certainty, as well as guiding interventions for those who will most benefit.
